# Beak shape and nest material use in birds

**DOI:** 10.1098/rstb.2022.0147

**Published:** 2023-08-28

**Authors:** Catherine Sheard, Sally E. Street, Caitlin Evans, Kevin N. Lala, Susan D. Healy, Shoko Sugasawa

**Affiliations:** ^1^ School of Earth Sciences, University of Bristol, Life Sciences Building, 24 Tyndall Avenue, Bristol BS8 1TQ, UK; ^2^ Department of Anthropology, Durham University, Dawson Building, South Road, Durham DH1 3LE, UK; ^3^ School of Biology, University of St Andrews, Harold Mitchell Building, St Andrews KY16 9TJ, UK

**Keywords:** object manipulation, form-function coevolution, bird nests, morphology, construction, behaviour

## Abstract

The evolution of behaviour can both influence, and be influenced by, morphology. Recent advances in methods and data availability have facilitated broad-scale investigations of physical form and behavioural function in many contexts, but the relationship between animal morphology and object manipulation—particularly objects used in construction—remains largely unknown. Here, we employ a new global database of nest materials used by 5924 species of birds together with phylogenetically informed random forest models to evaluate the link between beak shape and these nest-building materials. We find that beak morphology, together with species diet and access to materials, can predict nest-material use above chance and with high accuracy (68–97%). Much of this relationship, however, is driven by phylogenetic signal and sampling biases. We therefore conclude that while variation in nest material use is linked with that of beak shape across bird species, these correlations are modulated by the ecological context and evolutionary history of these species.

This article is part of the theme issue ‘The evolutionary ecology of nests: a cross-taxon approach’.

## 1. Introduction

The ability to manipulate objects can have significant evolutionary consequences, as animals can gain substantial fitness from successful object manipulation during foraging, transport or construction [1,2]. Indeed, skilled forelimb movements, including object manipulation, appear to have been acquired early in the tetrapod lineage [3,4], and the ability of primate hands to manipulate objects is considered a key innovation in that clade's evolutionary history [3,5,6]. For example, efficient tool-making capacity is linked to specific morphological characteristics in hands, such as broad fingertips or enlarged muscles in the thumb and little finger [7,8]. The evolutionary effects of these innovations, however, are confounded by multiple other selection pressures acting on limbs, including locomotion, and studying this phenomenon is hindered by the lack of standard behavioural descriptions that could be analysed in a comparative context [4]. Furthermore, far less is known about the evolutionary causes and consequences of object manipulation in animals that lack hands or paws, despite diverse examples of animals manipulating objects using other body parts such as mouths and fins [1,9].

Avian nest building provides a particularly useful example of construction behaviour without hands. Most (but not all) of the roughly 10 000 extant species of birds build nests, and there is considerable diversity in the structure of, and material used in, nests. Across bird species, nest structures range from simple scrapes on the ground to excavated cavities to elaborate, multi-chambered constructs [10,11], and the materials used to build nests include mud, grass, twigs, feathers, and artificial materials such as plastic [12–15]. The chief appendage birds use to build these nests is their beak, although some birds additionally use their body and feet [16,17]. An extensive body of literature spanning Darwin's descriptions of Galapagos finches [18] to modern phylogenetic studies has examined the link between interspecific variation in beak shape and diet [19–21], foraging strategy [19], song [22,23] and the environment [24,25]. The extent to which beak morphology correlates with species-level differences in nest-building materials, however, is currently unknown, beyond speculation that there is little to no relationship between the two [26]. This is surprising, given that nest building is crucial to an individual's reproductive success, with the interaction between a beak and nest materials key to forming the resulting structure of the nest [27]. Moreover, while behaviours such as nest-material selection have been widely assumed to be a particularly plastic and evolvable trait (e.g. [28]), beak shape is also highly evolvable [29,30], suggesting that these two traits may potentially coevolve.

Recent comparative work has begun to examine broad-scale correlates of the interspecific variation in nest structure (e.g. [31]), site (e.g. [32]), and size (e.g. [33]), but little is known about the evolutionary sources of interspecific variation in nest-material use itself. We here attempt to determine if the extensive species-level diversity in avian beak morphology can be linked to the wide variation in nest-material use, using flexible machine-learning algorithms known as random forest models. As beaks are ‘multi-purpose organs' [26] that are used for many different tasks beyond nest building, such as foraging, we anticipate that any potential form-function correlations are modulated to some extent by ecological variables. We first present a new database of nest materials for 5924 species of birds from 38 of 40 orders and 180 of 194 families. We then combine this data on nest materials with beak traits from AVONET [34], as well as information about species diet and proxies for material availability, to evaluate the associations between beak morphology and nest-material use across the class. We also distinguish between species that use a single type of material (nest-material specialists) and species that use multiple types of material (nest-material generalists). Finally, we use simulations to control for the shared evolutionary history between related species (phylogenetic signal) and for sampling biases.

## 2. Material and methods

### (a) Data collection

**Figure 1.**
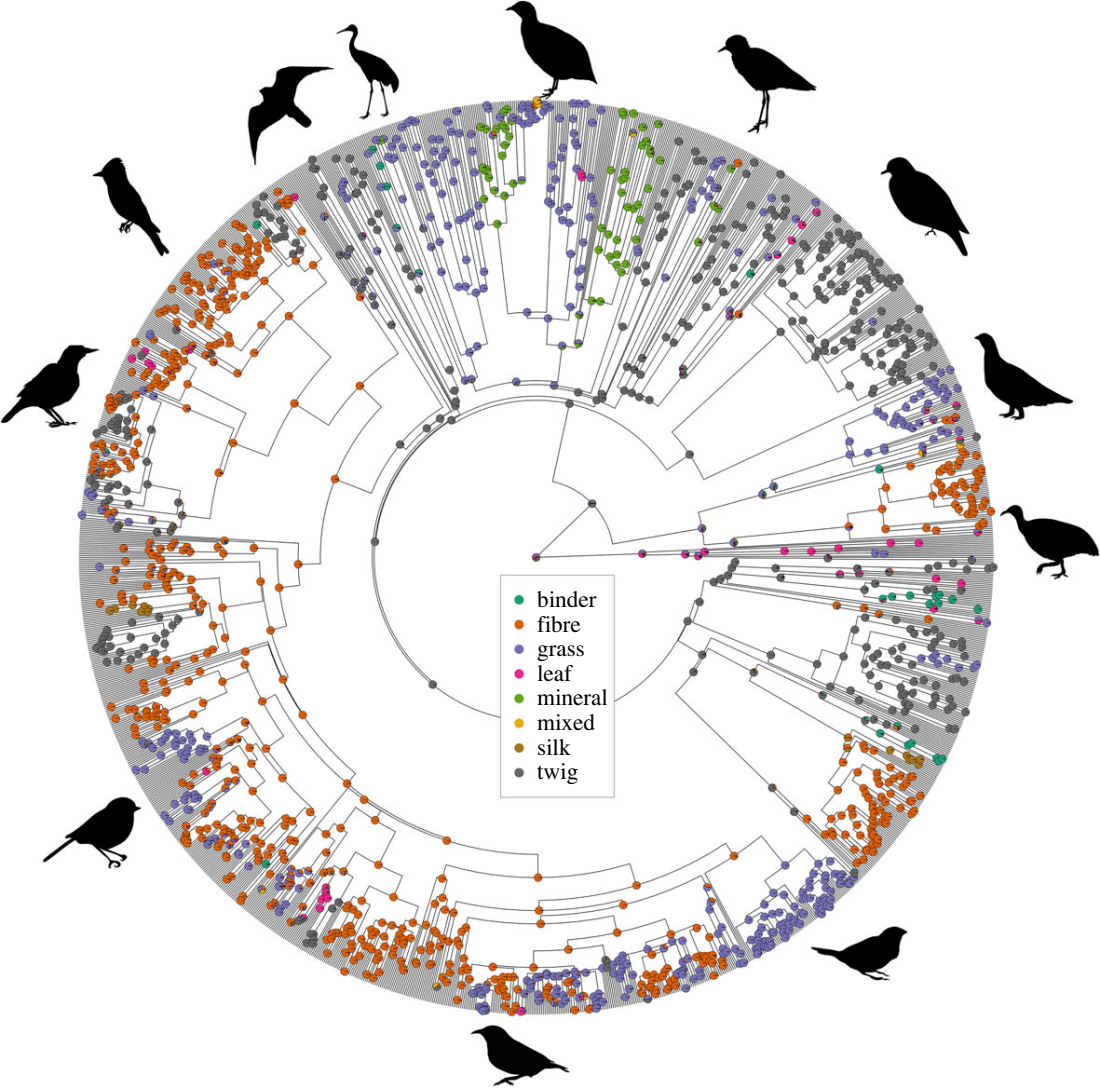
Phylogenetic distribution of material specialists. Shown for display purposes only is an arbitrary topology from the Global Bird Tree (Jetz *et al*. [37]) as well as an ancestral state reconstruction assuming equal transition rates calculated using the ‘ace’ function in the R package *ape* [38]. Illustrative silhouettes from phylopic.org indicate approximate phylogenetic positions; please see the electronic supplementary material for further information on image licensing.

Descriptions of nest materials were collated from the *Handbook of the Birds of the World Alive* (2017–2018), *Neotropical Birds Online* (2019–2020) and the *Birds of* *North America Online* (2019–2020), all of which sources have subsequently been combined into a single publication, the *Birds of the World* [35]. Each nest material used by each species was then scored as belonging to one of seven categories: binder (e.g. mud, sand, saliva, peat, droppings), fibre (e.g. feathers, fur, hair, plant down, moss, fern, fungi, rootlets), filamentous grass-like material (here termed ‘grass’, including stems, grassy vines, Spanish moss, seaweed, kelp, algae), leaf, mineral (e.g. shell, bones, pebble), silk or twig (e.g. wood, root, bark, liana, woody vines, heather). For each species, we also recorded what we term the ‘primary’ material from these seven categories, here defined as either the material category indicated in-text as the most commonly used; the only category reported; or, in absence of any other information, the category of the first material listed (following Wilman *et al*. [36] and Pigot *et al*. [19]). Species with different primary materials depending on the source consulted were considered ‘mixed’. We omitted from analysis species lacking data on material use, those that do not build nests, and those with ambiguous descriptions of material type (e.g. owing to scorer uncertainty when identifying materials from images, owing to contradictory information within a species entry, or owing to ambiguity between nest materials and substrate), leaving a total of 5924 species out of 9993 species with phylogenetic information (figure 1).

To capture key dimensions of beak size and shape, four linear morphological measurements were obtained from AVONET [34]: total bill length, bill length from the nares, bill width at the nares, and bill depth at the nares. Although these traits are intercorrelated (as well as correlated with other ecological variables included in these models), we left each variable untransformed in order to improve ecological interpretability. We also included the AVONET body mass measurement, to control for beak shape allometry as well as in part for the known correlations between body mass and life history.

To represent species diet, we used the three AVONET variables ‘trophic level’ (carnivore, herbivore, scavenger, or omnivore), ‘trophic niche’ (one of 10 dietary categories, e.g. invertivore, frugivore, granivore) and primary lifestyle, here termed ‘trophic lifestyle’ for consistency (aerial, aquatic, generalist, insessorial (perching), or terrestrial). As a proxy for access to materials (material access), we included three variables. First, we used two measures of habitat, one simple (‘habitat density’: dense, semi-open or open), and one complex (one of 11 categories, e.g. forest, desert, grassland). We assumed that different materials will be available in each of the different habitats listed. Second, we approximated material access using the AVONET variable of hand-wing index (HWI; a common proxy for flight ability, see [39]). We assumed that stronger long-distance fliers may have access to more types of material than short-distance fliers, and that different flight styles might face different predation pressures during the nest-building phase [40,41]. HWI is, however, also closely related to migratory behaviour, so to partition the flight ability variable from this relationship, we also included the AVONET variable of migration (sedentary, partially migratory, or migratory). Further details can be found in the electronic supplementary material, table S1.

### (b) Random forest models

To assess the relationship between beak shape and nest material, we considered three different types of response variable. First, we considered each material category as a separate binary variable (i.e. either that species is or is not known to use that material). Second, we used the categorical trait ‘primary material’ (i.e. the material category assigned as the most commonly used by each species) as the response variable. Third, we ran a set of models with only material specialists, or species scored as building using only one category. These were similar in structure to the primary material models, but on a reduced dataset. For each model, we ran random forest models using the R package *randomForest* [42] in R v. 4.1.3 [43] using 5000 decision trees; random forest models were selected to account for the complex, nonlinear structure of the data [44] and generally follow the same basic analytic outline as in [19]. In brief, random forest models are a type of machine learning algorithm that use the input variables to create decision trees to predict a categorical classification. We first ran a naive set of models with only the beak morphology traits as predictor variables to establish a baseline estimate of the correlation between these two suites of traits. We then included all potential predictor variables—four measures of beak morphology, three measures of species diet, and four measures of material access—to address the central questions of this study. To provide an estimate of the strength of each predictor within these models, we then ran versions of each model with each predictor variable removed (marginal contribution) and versions of each model with only one of each predictor variable included (independent contribution).

There are two major sources of bias in this modelling approach. The first is phylogenetic inertia: similarity in the response and predictor variables could be the result of shared evolutionary history rather than a true ecological relationship. To evaluate the effect of this phenomenon, for each of the six continuous and six discrete predictor variable in our models (see the electronic supplementary material, table S1), we first generated simulated data using 100 topologies randomly selected from the Hackett backbone of the Global Bird Tree [37] and realistic Brownian motion parameters, calculated with the ‘fitContinuous’ and ‘sim.char’ commands in the R package *geiger* [45]. We then re-ran the random forest models on these simulated datasets. A prediction success rate on the simulated data that is close to that of the observed data would indicate high phylogenetic signal in this modelling approach; a low prediction success rate on the simulated data would indicate that shared evolutionary history is playing little role in driving the observed ecological correlations. Further information on the phylogenetic simulations can be found in the electronic supplementary material.

The second source of bias is the asymmetry of the data: some material categories occur more frequently than others, and thus a model could be achieving apparent success by simply guessing the common categories and ignoring the rare ones. To evaluate the extent to which this strategy may be driving the reported model results, for each model we also present the predictive power of a ‘downsampled’ version, where species from more frequent response categories are omitted from the model at random until an equal number of species remain for each category. The results we present are the median from 100 such downsampling attempts. In this case, a prediction success rate within the downsampled data close to that of the observed data would indicate that the frequency of the materials plays little role in the model outcome.

To improve reproducibility in the simulations, all models are preceded by a ‘set.seed’ function, but all such six-digit seeds were generated randomly.

### (c) Phylogenetic generalized linear models

To provide phylogenetically corrected estimates of the relationships between nest-material use and the 12 morphological and ecological variables considered here, we also ran a set of phylogenetic generalized linear models (GLMs) using the function ‘phyloglm’ in the package *phylolm* [46]. These GLMs could only be used for binary response variables (as opposed to the random forest models, which provide comparable structures for both binomial and multinomial responses), and unlike random forest models assumes a linear relationship within the logit link.

**Figure 2.**
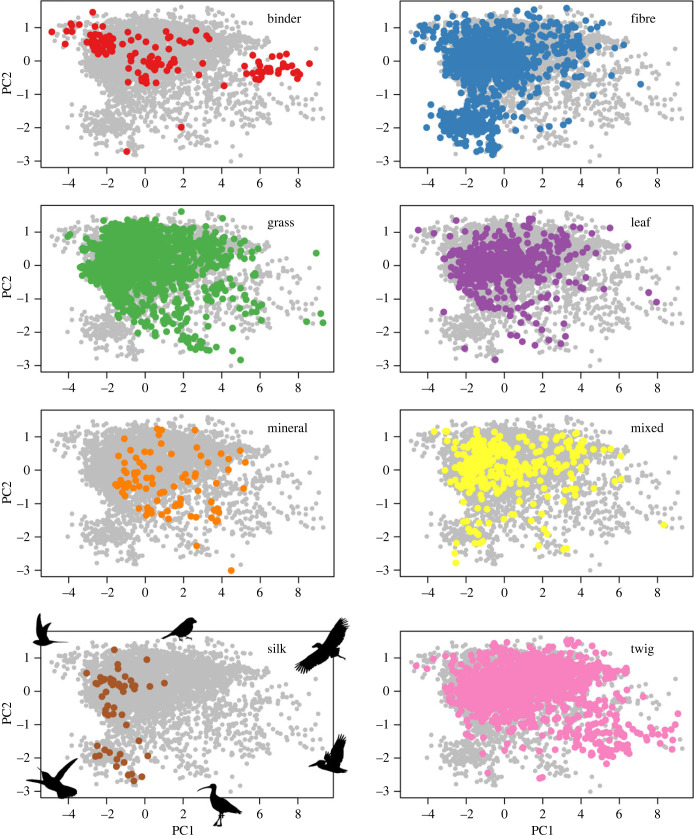
Partitioning of beak morphospace by primary nest material. Within each panel, coloured dots denote species with the listed primary nest material; grey dots denote all other species. Axes represent the first (*x*-axis) and second (*y*-axis) principal component of a morphospace formed of the four beak morphology variables and body size. PC1 is generally a measure of bill size, ranging from swiftlets (lowest) to storks and pelicans (highest); PC2 is a ratio of width/depth to length, ranging from the long-billed curlew (lowest) to wood-partridges and frogmouths (highest). Illustrative silhouettes from phylopic.org on the ‘silk’ panel indicate approximate morphospace positions; please see the electronic supplementary material for further information on image licensing.

To reduce multicollinearity among the predictor variables, we first ran a principal components analysis on the four beak morphology variables together with body size using the function ‘prcomp’ (figure 2, where these traits are also included for visualization purposes) and included the first two variables (principal component 1 (PC1), broadly interpretable as size; and principal component 2 (PC2), broadly interpretable as shape) in our models; we also ran model selection procedures to determine the models of best fit, based on Akaike information criterion (AIC) fit, with ΔAIC > 2 considered statistically meaningful. All models were run on a consensus tree computed from the 100 topologies used in the phylogenetic simulations, using TreeAnnotator [47], and all continuous variables were scaled to have a mean of 0 and variance of 1 to improve coefficient interpretability.

## 3. Results

Random forest models indicate a link between beak morphology and nest-material use: the four beak shape variables, together with body mass, can correctly predict primary nest-material use in 48.4% of cases (rising to 59.8% when restricted to material specialists and with the ecologically meaningless ‘mixed’ category omitted), as well as individual material use from 63.8% of cases (leaf) to 96.8% of cases (mineral) (electronic supplementary material, table S2). The addition of alternative drivers and confounding ecological effects (i.e. measures of diet and material availability) further increase the predictive power of these models, with primary nest material correctly predicted 56.0% of the time (rising to 70.0% of the time within material specialists and with the ‘mixed’ category omitted; electronic supplementary material, table S2), and with individual material use correctly predicted between 68.8% of the time (leaf) and 97.2% of the time (mineral) (table 1).

**Table 1.** Model predictive power of individual nest materials. (All models were run on a sample size of 5924 species. The models considered each material as a binary (i.e. either a species is known to use that material, or it is not) and used the 12 morphological and ecological variables to assign each species to a category. High model accuracy rates (close to 100) indicate models able to successfully predict nest-material use based on the species' morphological and ecological traits; low accuracy rates (close to 0) indicate low success. Phylogenetic simulation accuracy rates indicate success rates on simulated data; the higher this number, the more likely it is that shared evolutionary history is biasing these models. Downsampled predictive power indicates model accuracy results on subsamples with equal category sizes; in this column, values close to the overall accuracy rate indicate low levels of frequency bias.

material	sample size	accuracy rate	phylogenetic simulation accuracy rate	downsampled predictive power
binder	409	94.7	93.6	78.7
fibre	3989	79.3	76.2	73.9
grass	3661	72.3	68.3	70.2
leaf	2185	68.8	66.6	65.5
mineral	186	97.2	96.9	85.2
silk	991	85.5	84.6	79.2
twig	2776	71.5	66.4	70.9

**Figure 3.**
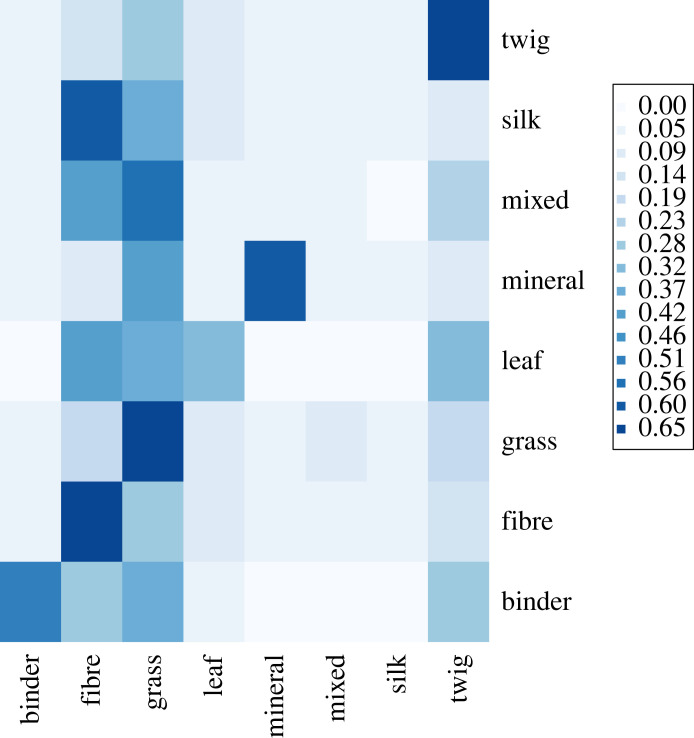
Confusion matrix for the primary material model, including beak morphology and all ecological co-variates as predictors. Rows represent observed material; columns represent model-assigned guesses. Dark blue indicates popular guesses for that row (i.e. a larger proportion of guesses for a given observed material), while white or light blue indicates rare guesses. The category ‘mixed’ indicates mixed evidence for primary material between sources (*n* = 331). See the electronic supplementary material, table S3 for total values.

Our main model considers the ability of beak morphology and the additional covariates controlling for species diet and access to material to correctly predict species-level primary material use, out of eight potential choices (figures 2 and 3; electronic supplementary material, tables S3–S4). This model was correct in 56.0% of cases and was most successful at predicting grass use (69.2% success) and least successful at silk use (0% success). Many categories were frequently mis-identified by the model as grass or fibre, suggesting a possible sampling bias, and both binder and leaves were further frequently mis-identified as twigs. Although the ‘mixed’ category, denoting species where the primary material scoring differed between sources, has little ecological meaning, removing it from the model only slightly improved accuracy to 59.7% (electronic supplementary material, figure S1, table S5).

Among specialists, random forest models were able to predict nest material 68.5% of the time (electronic supplementary material, figure S2, tables S6–S7), or 70% of the time with the ‘mixed’ category omitted (electronic supplementary material, figure S3, table S8). Within this sample, the models were most successful at predicting fibre (82.2% success), although again least successful with silk (0% success).

These predictor variables are, however, inter-correlated, and thus extracting an independent correlation between a single variable and the model success is difficult. Across all species, HWI (a proxy for flight ability, [48,49]), body mass and habitat type had the largest independent contributions to models predicting primary nest material (electronic supplementary material, table S4), but said roles were relatively modest. Among nest-material specialists, the most independently informative variable was trophic lifestyle (i.e. whether a species was aerial, aquatic, generalist, insessorial or terrestrial; electronic supplementary material, table S7), although, again, this value represented a small marginal contribution. Models predicting specific material use indicate a somewhat larger independent contribution of body mass and HWI, though with generally low predictive values overall (electronic supplementary material, tables S9–S15); this probably reflects the high correlations among the other predictor variables, such as between the beak shape and dietary traits (e.g. [19,39] for more information on how these variables relate to one another).

While the direction and magnitude of these effects are difficult to assess because of the structure of the random forest models, in general, among the trophic lifestyles, aerial species use binder a disproportionate amount, while insessorial (perching) species disproportionately use fibre and terrestrial species disproportionately use grass (electronic supplementary material, figure S4). Species with high HWI (high flight ability) tend to use minerals and species with low HWI (low flight ability) tend to use leaves, with silk and binder use found in species with very broad ranges of flight ability (electronic supplementary material, figure S5). Species that use twig, binder or mineral as primary material also tended to be larger, while species that use silk as a primary material tend to be very small (electronic supplementary material, figure S6).

It is important to note, however, that there are two major sources of bias inherent in this modelling approach. Phylogenetic simulations suggest that 66.4–96.9% of interspecific variation in individual nest-material use (table 1), as well as 48.6% of variation in primary nest material (electronic supplementary material, table S4) and 56.6% of the variation among the material specialists (electronic supplementary material, table S7), can be attributed to phylogenetic signal under Brownian motion. (For comparison, about 65% of the variation in the main model of [19] could be attributed to phylogenetic simulations, and the marginal *R*^2^ of phylogenetic generalized linear mixed models in other avian traits can be as high as 0.95 [50] or 0.36 [51].) Furthermore, the models predicting primary material and material specialism were partially dependent on frequency bias; downsampled models were only able to predict 43.6% (primary material, electronic supplementary material, table S4) and 47.8% (material specialism, electronic supplementary material, table S6) of the interspecific variation. The models predicting individual material use were somewhat less sensitive to frequency bias, with all down-sampled models scoring at least 65% accuracy (table 1).

Phylogenetic GLMs broadly confirm our findings that beak size and shape correlate with, but are not the main drivers of, nest-material use. Beak size (PC1) and shape (PC2) were identified in model selection procedures as significant correlates of the use of binder, fibre, grass (shape only), silk (size only) and twig, but not leaf or mineral (electronic supplementary material, tables S16–S23).

## 4. Discussion

Here, we demonstrate that variation in beak shape and nest material can be linked across a large sample of birds, but that these relationships are modulated by ecological co-variates, phylogenetic signal and frequency bias. For example, while grass is used by birds with many different beak sizes and shapes (other than small, pointy ones), silk is generally only used by small-billed species (of any width/depth: length ratio), and binder is generally only used by species with especially bulky bills (of any size). Given the complexity of these relationships, this is not necessarily evidence of co-evolution (i.e. evolutionary changes between traits that affect each other reciprocally). That such relationships exist at this interspecific scale, however, even after controlling for many forms of bias in the machine-learning approach, indicates an under-appreciated link between selection for object manipulation and broad-scale variation in beak shape. Beaks are a multi-purpose tool [24], and for those species that build a nest, that nest is a key component of avian reproduction and parental care [2,11,52]; we here establish a correlation between these two suites of traits.

Avian beaks serve many ecological purposes, with variation in beak size and shape previously associated with, for example, diet, foraging strategy, song and environmental gradients [19–25,53]. Indeed, some of these previously demonstrated correlations seem to conflict with each other, in part apparently dependent on the phylogenetic scale analysed (e.g. class [19] versus order [20,53]) and/or on which co-variates are included in the modelling approach (e.g. [23]). The potential evolutionary causes and effects of variation in beak morphology are therefore not straightforward; for each of these correlations, it could be that selection acting on one relationship is guiding another, or that some of these correlates of beak shape are themselves co-evolving. While we have controlled for some ecological co-variates within our models, no single study can capture all possible alternative drivers of both beak morphology and nest-material use, particularly not in a sample where the phylogenetic signal is so high. Instead, we offer this broad-scale correlation as a first step, and call on other studies, at many different levels, to establish the exact evolutionary mechanisms driving this pattern. For example, comparative studies of beak shape and diet have begun to explicitly include biomechanical data on the physical properties of the beak (e.g. [21,54]), while a clearer understanding of how different bill morphologies interact with different nest materials could highlight biologically relevant aspects of nest materials that could then inform future comparative work. Material rigidity, for example, could together with individual preferences be linked with zebra finch (*Taeniopygia guttata*) fledgling success in an experimental setting [2]; it could be that different bill shapes are better adapted for manipulating materials with different optimum rigidities.

Although nest structure and location are clearly shaped by natural selection [40,41], nest-material choice is sometimes considered a plastic and opportunistic trait [2,11]. In pied flycatchers, for example, nest-material choice varies with material availability [55], while cigarette butts are incorporated into the nests of house sparrows (*Passer domesticus*) and house finches *(Haemorhous mexicanus*) at a greater rate in urban environments [56], and the incorporation of wool into great tit nests correlates with the proximity of sheep [57]. Other material preferences, however, seem to be more stable [58,59], and might be driven, for example, by sexual selection or by selection for particular thermal or anti-predator properties [33,60–63]. Moreover, the correlations that we find here, over and above the effects of phylogenetic signal and sampling biases, do indicate that interspecific variation in the beak shape and in coarse nest material preferences can be linked. Furthermore, the confusion matrices of our models may indicate which broad categories of material could fulfil similar ecological roles; for example, while the models were frequently able to predict which species used twigs as a primary material, there was less predictive success with leaves (e.g. figure 3). It thus may be that twigs serve a more mechanically distinct role in the nest structure than do leaves, and thus the incorporation of twigs into nests might be less plastic (or less evolvable) than the incorporation of leaves. Indeed, this may even suggest a potential link between beak morphology and other aspects of nest design, such as nest structure.

We find that body mass and HWI (an index of flight ability and common proxy for dispersal) are consistently meaningful predictors of interspecific variation in nest-material use. The relationship between body mass and nest material may reflect energetic constraints (e.g. that only larger birds can carry and manipulate heavier materials) and/or the need for the nests of larger birds to maintain structural integrity via stronger materials [15,64,65]. Across a sample of 591 passerine species, heavier birds are known to use more woody material in the outside of the nest [64], but otherwise little is known about how mass relates to nest construction on a comparative scale. The relationship with HWI is less easily interpreted. It could be that birds of different flight abilities have different access to materials (e.g. if the association between high HWI and mineral material usage reflects the need to fly farther to obtain suitable pebbles); there is an energetic cost of nest building [66], and many individuals may build nests by opportunistic material gathering [55]. Alternatively, as HWI tends to be lower in tropical species [39], which in turn tend to be under-studied [67], this result could reflect bias in research effort. If, for example, tropical birds were more likely to be identified as nest-material specialists based on under-documentation rather than true representation of nests, the machine-learning algorithms could be using a small HWI to produce this hypothetical trend rather than predicting a real ecological relationship.

Outside the primates, very little is known about the role of object manipulation as either a cause or a consequence of morphological variation. In a global sample of birds, we have here established a strong correlation between species-level nest-material use and beak morphology, highlighting that form-function co-evolution can occur even in a relatively variable trait. This relationship, however, is tempered by underlying phylogenetic and sampling biases. Further comparative data on how specific beak measurements translate to nest-building efficiency, as well as on the physical role of different nest materials in structuring a nest [15], will clarify the evolutionary links between nest-material choice and beak shapes.

## Data Availability

The data are included in an Excel file as the electronic supplementary material [68].
